# *INMAP* Overexpression Inhibits Cell Proliferation, Induces Genomic Instability and Functions through p53/p21 Pathways

**DOI:** 10.1371/journal.pone.0115704

**Published:** 2015-01-30

**Authors:** Yan Zhu, Yan Lei, Baochen Du, Yanbo Zheng, Xiangfeng Lu, Tan Tan, Jingting Kang, Le Sun, Qianjin Liang

**Affiliations:** 1 Beijing Key Laboratory of Gene Resource and Molecular Development, College of Life Sciences, Beijing Normal University, Beijing, 100875, P. R. China; 2 Key Laboratory of Cell Proliferation and Regulation Biology of Ministry of Education, College of Life Sciences, Beijing Normal University, Beijing, 100875, P. R. China; 3 The Institute of Medical Biotechnology (IMB) of the Chinese Academy of Medical Sciences, Beijing, 100050, P. R. China; 4 Dongzhimen Hospital of Beijing University of Chinese Traditional Medicine, Beijing, 100700, P. R. China; 5 AbMax Biotechnology Co., Haidian, Beijing, 100085, P. R. China; Tel-Aviv University, ISRAEL

## Abstract

INMAP is a spindle protein that plays essential role for mitosis, by ensuring spindle and centromere integrality. The aim of this study was to investigate the relevant functions of INMAP for genomic stability and its functional pathway. We overexpressed *INMAP* in HeLa cells, resulting in growth inhibition in monolayer cell cultures, anchorage-independent growth in soft agar and xenograft growth in nude mice. In this system caused micronuclei (MNi) formation, chromosome distortion and *γH2AX* expression upregulation, suggesting DNA damage induction and genomic stability impairment. As a tumour biochemical marker, lactate dehydrogenase (LDH) isoenzymes were detected to evaluate cell metabolic activity, the results confirming that total activity of LDH, as well as that of its LDH5 isoform, is significantly decreased in *INMAP*-overexpressing HeLa cells. The levels of p53 and p21 were upregulated, and however, that of PCNA and Bcl-2, downregulated. Indirect immunofluorescence (IIF) and coimmunoprecipitation (CoIP) analyses revealed the interaction between INMAP and p21. These results suggest that INMAP might function through p53/p21 pathways.

## Introduction

INMAP (interphase nucleus/mitotic apparatus protein) is a 38.2-kDa conserved protein and plays vital roles in spindle assembly and cell proliferation. Abnormal expression of *INMAP* leads to mitotic aberration, malignant cell proliferation or apoptosis. Our previous studies revealed that stable overexpression of *INMAP* in HeLa cells causes the formation of abnormal mitotic spindles, decreased DNA content and split intracellular vesicles, thereby brings out cell-cycle arrest and apoptosis[1]. INMAP deficiency, even though not producing gross defects in spindle formation, affects chromosome segregation, and, more significantly, regulates mitosis through modulating the CENP-B-mediated centromere organisation, by rendering CENP-B cleavagable, and induces the centromeric halo reflecting unstable falling apart centromeres [2]. These results showed that an appropriate INMAP level is physiologically necessary, abnormal level affecting the fate of cells.

p21 is a key factor regulated by p53 in response to DNA damage [3, 4], accumulating in cell nucleus owing to increasing gene expression after DNA damage. It binds to CDKs and suppresses their activity, leading to cell-cycle arrest at the G1/S or G2/M phase [5]. Cell-cycle arrest induces the function of p21 in promoting error-free replication-coupled DNA double-strand-break (DSB) repair [6], as well as inhibiting DNA replication by binding with the proliferating cell nuclear antigen (PCNA), DNA polymerase-δ and several other proteins involved in DNA synthesis [6–8]. In addition, p21 can promote apoptosis through both p53-dependent and p53-independent mechanisms under certain cellular stresses, inducing upregulation of the pro-apoptotic protein BAX and activation of tumour necrosis factor family members of death receptors [9].

In a recent study, we detected the effect of *INMAP* overexpression in HEK293T cells, revealing that high level of INMAP represses *p53* and *AP-1* (activator protein-1) transcriptional activity in a dose-dependent manner [10]. Therefore, biological activity of INMAP may be related to carcinogenesis through p53 and AP-1 pathways. It is clear that INMAP interacts with proteins such as NuMA (Nuclear Mitotic Appratus protein) [1], a protein required for the selective induction of p53 target genes and playing a crucial role in regulating p53 mediated transcription in response to DNA damage. Following DNA damage, the level of the NuMA-p53 interaction gradually increases in a time-dependent manner. Binding to CDK8, NuMA also activates the downstream gene *p21* and causes cell-cycle arrest. The ablation of NuMA attenuates the pro-arrested *p21* gene induction following DNA damage, and consequently, cell-cycle arrest is impaired [11]. Notably, the clear determination on whether and how the functions of INMAP are involved with p53 signalling pathway is ponderable.

The goals of this study were to assess whether a high level of INMAP may affect tumour growth and to explore the functional pathway of INMAP. We constructed a HeLa cell experimental model with stable overexpression of *INMAP* and analysed the frequency of micronuclei and degree of chromosome distortion induced by *INMAP* abnormal expression. Associated with this, cell growth ability in monolayer cultures, soft agar culture medium and implanted nude mice were detected. Furthermore, we studied the changes in expression of several key proteins in p53-mediated pathways. The results provide evidences that overexpression of *INMAP* inhibits tumour growth through the p53/p21 signalling pathways.

## Materials and Methods

### Ethics Statement

All animal experiments described in this study were approved by the Animal Care and Use Committee of Peaking University Health Science Center (PUHSC). The experiments were performed in strict accordance with guidelines of International Association for the Study of Pain.

### Cell lines, cell culture and antibodies

HeLa cells were cultured in DMEM (Dulbecco’s modified Eagle medium, Invitrogen, USA) supplemented with 10% FBS (fetal bovine serum, Invitrogen, New Zealand), 100 U/mL penicillin and 100 mg/mL streptomycin at 37°C with 5% CO_2_. Some of these cells were transfected with p3XFlag-CMV14 empty plasmid vector (Flag-HeLa) and p3XFlag-*INMAP*-CMV14 recombinant vector (Flag-INMAP) [10], respectively. Flag-HeLa and Flag-INMAP cells were cultured in DMEM with 600 ng/μL geneticin G418 (Merck, USA). The expression of *INMAP* was detected in stable single cell clones using a Flag monoclonal antibody and an INMAP polyclonal antibody.

Several mouse monoclonal antibodies, including anti-Flag (MBL, Japan) anti-His (MBL, Japan) and anti-GAPDH (MBL, Japan) antibodies, rabbit monoclonal antibodies including anti-p21 (CST, USA), anti-p53 (CST, USA), anti-γH2AX (Bioworld, USA), anti-Bcl-2 (Santa Cruz, USA) antibodies and mouse polyclonal anti-INMAP (Beijing Normal University, China) antibody were used in immunoblot, immunoprecipitation and immune fluorescence experiments. Mouse monoclonal anti-PCNA antibody was provided by Dr. Jian Kuang (University of Texas M. D. Anderson Cancer Center, USA). TRITC-conjugated goat anti-rabbit IgG and FITC-conjugated goat anti-mouse IgG were obtained from Vector Laboratories (Peterborough, UK).

### Colony formation assay

Cells (n = 100) were seeded into each well of 12-well plates and cultured in 1 mL of DMEM with 10% FBS. After incubation for 7 d, cells were rinsed with PBS (phosphate-buffered saline) 3 times, fixed in 100% methanol for 15 min and washed with distilled water. Plates were stained for 30 min in the dark with 10% Giemsa stain. Colonies with more than 50 cells per colony were counted. All of the experiments were performed in 4 wells in 3 independent experiments.

### Anchorage-independent cell growth in soft agar

Wells of 12-well plates were coated with 1 mL of bottom agar mixture (DMEM with 10% FBS and 0.6% agar). After the bottom layer had solidified, 1 mL of the top agar mixture (DMEM with 10% FBS and 0.3% agar) containing 2 × 10^3^ cells was added. The medium over the agar was replaced every 4~5 d or as needed. After 5 weeks of growth, colonies (≥50 cells/colony) were counted under an optical microscope.

### Western blot analysis

Proteins were extracted with RIPA buffer (Roche, USA), and the concentrations were determined with a BCA kit (Vigorous, China). Samples consisting of 30 μg of proteins were loaded in each lane, resolved via 12% SDS-PAGE (sodium dodecyl sulphate polyacrylamide gel electrophoresis), and transferred to nitrocellulose (NC) membranes. Membranes were blocked in TBST (Tris-buffered saline with 0.1% Tween 20 solution) containing 5% skim milk for 2 h at room temperature (RT) and incubated with primary antibodies overnight at 4°C. Membranes were then washed 3 times in TBST and incubated with peroxidase-conjugated secondary antibodies for 1 h at RT. Blotted proteins were detected using an ECL detection system (Vigorous, China) with Kodak BioMix film and analysed with Image J software (http://www.uhnresearch.ca/facilities/wcif/download.php).

### Tumour Xenograft Model in Nude Mice

Nude mice were provided by the Department of Experimental Animal Sciences, Peking University Health Science Center. Six-week-old female Balb/c nude mice (5/group) were used for tumorigenesis assays. The right flank of each mouse was injected with 6 × 10^6^ cells. Every 4 d, tumour sizes were measured with a vernier caliper, and body weights were recorded. Tumour volume (cm^3^) was estimated by measuring the longest and shortest diameters of the tumour and calculated as described [12]. After 40 d, the mice were euthanised with 100% carbon dioxide inhalation, cervical dislocation followed. The tumours were removed and weighed. Simultaneously, to analyse the histopathological phenomena, the tumours and the liver of each mouse were stored in 4% paraformaldehyde, dehydrated in a graded ethanol series, embedded in paraffin, stained with haematoxylin and eosin (H&E), sectioned (5-μm thick), and observed with an optical microscope.

### Micronucleus assay

Cells were cultivated in 6-well plates. After 24 h, the medium was removed. The cells were washed with PBS 3 times, fixed in 100% methanol for 15 min, washed with distilled water, and stained for 30 min in the dark with 10% Giemsa stain. The micronuclei with diameters of less than 1/3 of the main nucleus in the same cell were counted. This experiment was conducted 3 times, independently, with 400 cells analysed in each replicate to evaluate the frequencies of micronucleated cells and micronuclei.

### Chromosome aberration assay

Cells were seeded into 75 cm^2^ flasks and treated with colchicine (final concentration 0.05 μg/mL) for 4 h. Then, the medium was removed, and the cells were incubated with a hypotonic solution (75 mM KCl) for 30 min at 37°C and centrifuged (70 × g) for 10 min at RT; the supernatant was discarded. Next, the cells were fixed using fixative solution (acetic acid: methanol, 1: 3) for 30 min at 4°C, and the supernatant was discarded again. The cells were suspended in 500 μL fresh fixative solution, placed on a clean glass slide, stained with Giemsa solution. We analysed 100 cells with chromosomes clearly in metaphase.

### Electrophoresis of LDH (lactate dehydrogenase) isoenzymes

Cells were harvested and washed twice with and suspended in PBS (pH7.2), then subjected to sonication for 10 times, each time for 3 s with an interval of 30 s, in an ice bath. Then, the homogenate was centrifuged at 10,000 × g for 20 min, and we collected the supernatant. LDH isoenzymes were separated by 7.5% PAGE and detected with 30 mL staining solution containing 15 mg nicotinamide adenine dinucleotide, 9 mg nitrotetrazolium blue chloride, 0.6 mg phenazine methosulphate, 1.6 mL of 0.1 M NaCl, 4.4 mL of 0.5 M Tris-HCl, and 380 μL dl-lactic acid sodium salt solution for 30 min at 37°C.

### Indirect immunofluorescence, co-immunoprecipitation and Pulldown assays

The methods for IIF, CoIP [1] and Pulldown [2] were described in our previous publication.

### Statistical analysis

Experiments were performed at least three times with duplicates. The data were analysed with one-way analysis of variance (ANOVA). All analyses were conducted with SPSS v19.0. Values of *P* < 0.05 were considered statistically significant.

## Results

### Overexpression of *INMAP* in HeLa cells

Quantitative analyses of INMAP expression was detected using an INMAP polyclonal antibody in HeLa, Flag-HeLa, Flag-INMAP cells, the result was shown in S1 Fig. INMAP expression was shown to be 2.82 ± 0.85 (means ± standard deviation, *P* = 0.011) fold and 1.03 ± 0.64 (*P* = 0.954) fold higher in Flag-INMAP and Flag-HeLa cells than that of in HeLa cells, indicating INMAP was stable expressed in Flag-INMAP cell line.

### Overexpression of *INMAP* reduces cell proliferation and anchorage-independent growth *in vitro*

**Figure 1:**
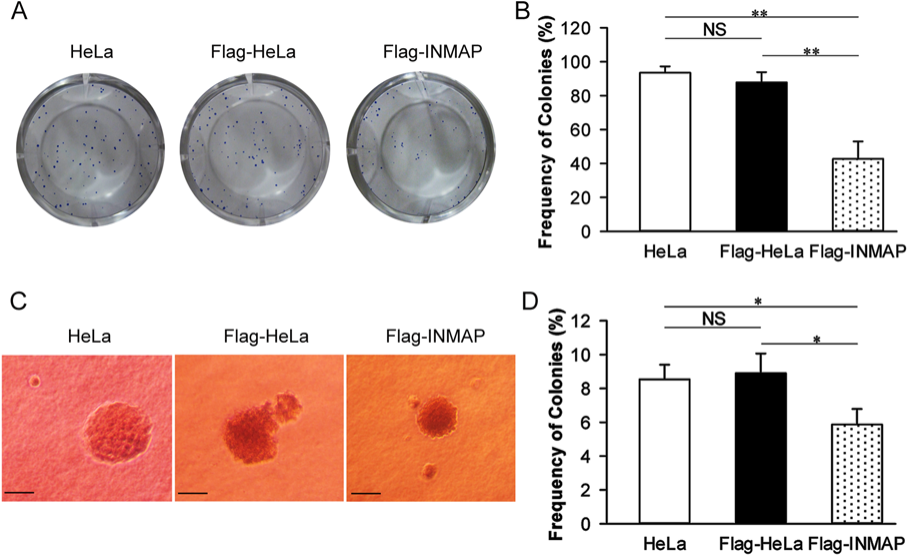
*INMAP* overexpression suppressed the growth of HeLa cells. (A, B). Colony formation of HeLa cells in monolayer cultures. Cells were stained with 10% Giemsa stain. Data are presented as mean ± SD; n = 4. ** and * represent significant differences with P-values under 0.01 and 0.05, respectively (the same below). NS, not significant. (C, D). Anchorage-independent colony growth assay in soft agar. Cells were seeded in 12-well plates containing 0.3% soft agar. After 6 weeks, the number of colonies with more than 50 cells was counted. Frequency of colonies of HeLa cells stably overexpressing *INMAP* in soft agar was analysed. Scale bars, 50 μm.

Anchorage-dependent growth in monolayer culture assays and anchorage-independent growth in soft agar analysis are extensively applied to detect the ability of cells to grow *in vitro* [13–16]. As shown in Fig. 1A and B, the frequency of colonies in monolayer cultures of *INMAP*-overexpressing cells (42.81% ± 10.23%) significantly decreased (*P* = 0.000) compared with Flag-HeLa (87.84% ± 6.03%) and HeLa (93.56% ± 3.69%). This showed that *INMAP* overexpression dramatically inhibits cell proliferation in monolayer cultures.

To examine the manner of cell growth in an anchorage-independent condition, we monitored the frequency of colonies in soft agar after 6 weeks in culture. Although HeLa cells grew well in soft agar, as shown Fig. 1C and D, the frequency of colonies in soft agar was reduced in *INMAP* overexpressing cells (5.87% ± 0.92%) compared with Flag-HeLa (8.91% ± 1.17%, *P* = 0.025) and HeLa cells (8.54% ± 0.87%, *P* = 0.044).

These results suggested that overexpression of *INMAP* inhibits the proliferation of HeLa cells *in vitro*.

### Inhibition of tumorigenesis by overexpressing *INMAP* in nude mice

**Figure 2:**
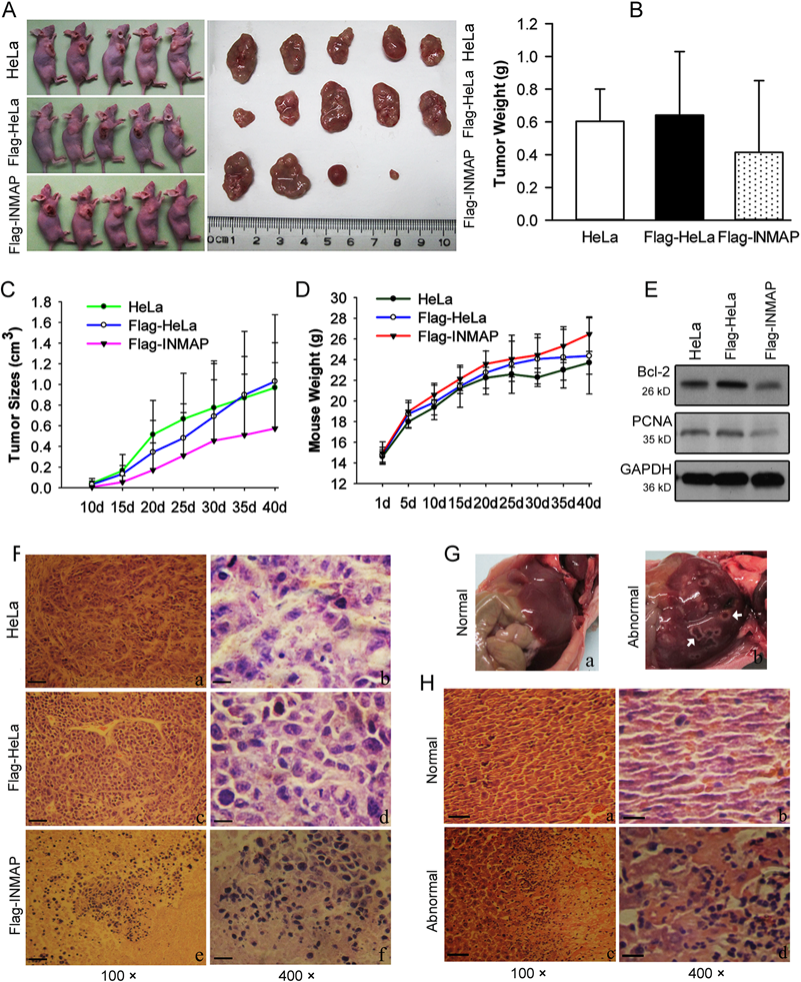
*INMAP* overexpression inhibited tumour growth *in vivo*. (A). Control (HeLa and Flag-HeLa) and overexpressing *INMAP* (Flag-INMAP) cells were injected into Balb/c nude mice (n = 5) in the back (near right axillary). 40 d later, the tumour xenografts were stripped. (B-D). Average tumour weights and sizes and mouse body weights were determined, respectively. No significant differences were detected. (E). Western blot analysis of the tumour tissues with anti-PCNA, anti-Bcl-2 antibodies. GAPDH was used as an internal control. (F). The histopathological analysis of the tumour tissues from different groups with H&E staining. Scale bars, 20 μm (a, c, e) and 5 μm (b, d, f). Larger necrotic areas were found in *INMAP*-overexpressing tumour tissue. (G). Morphological characteristics of normal (a) and abnormal (b) livers in nude mice. Inflammatory areas of the liver are labelled with arrowheads. (H). The histopathological analysis of normal (a, b) and abnormal (c, d) livers. Multiple necrotic areas, strongly stained nuclei, and infiltrated neutrophils were observed in abnormal livers. Scale bars, 20 μm (a, c) and 5 μm (b, d).

We investigated the effect of overexpressing *INMAP* on tumorigenesis in nude mice. On the fortieth day after cells were injected into nude mice, tumours were found in 5/5 HeLa or Flag-HeLa injected mice and in 4/5 Flag-INMAP injected mice (Fig. 2A). *INMAP* overexpressing cells grew into smaller tumour masses than control cells. The mean tumour weights were 0.60 ± 0.20 g (HeLa), 0.64 ± 0.39 g (Flag-HeLa) and 0.41 ± 0.44 g (Flag-INMAP), showing no significant difference (Fig. 2B). The *in vivo* subcutaneous tumour growth curve of HeLa stably transfected with *INMAP* or empty vector is shown in Fig. 2C. The mean tumour volume is lower in Flag-INMAP transfected nude mice compared to the control (HeLa or Flag-HeLa implanted) mice. There was no significant difference in the average weights of the mice (Fig. 2D).

Western blot assay and histopathological analysis of the tumour tissues revealed that overexpression of *INMAP* induces cell death, leading to suppression of tumour progression. *PCNA* and *Bcl-2* are downregulated in *INMAP*-overexpressing tumour tissues (Fig. 2E). H&E (Haematoxylin and eosin) staining showed that cell density decreased and disrupted cell morphology increased in the tumour sections from mice implanted with *INMAP*-overexpressing cells (Fig. 2F). The nuclei displayed more aberrant phenomena, such as pyknotic nuclei, nuclear fragmentation, and karyolysis. A low frequency of mitotic figures and larger necrotic area were observed. These results indicated that *INMAP* overexpression takes a tumour-suppressing effect on HeLa cells.

We noted two abnormal livers (Fig. 2G b) among the mice injected with standard HeLa cells and Flag-HeLa cells, but no abnormal liver was detected from mice injected with Flag-INMAP cells. Histopathologic examinations revealed that liver abnormalities were caused by inflammation. In the abnormal group (Fig. 2H c, d), the liver tissue shows typical pathological changes, multiple necrotic areas, strongly stained nuclei and infiltrating neutrophils.

### Overexpression of *INMAP* induces genomic instability in HeLa cells

**Figure 3:**
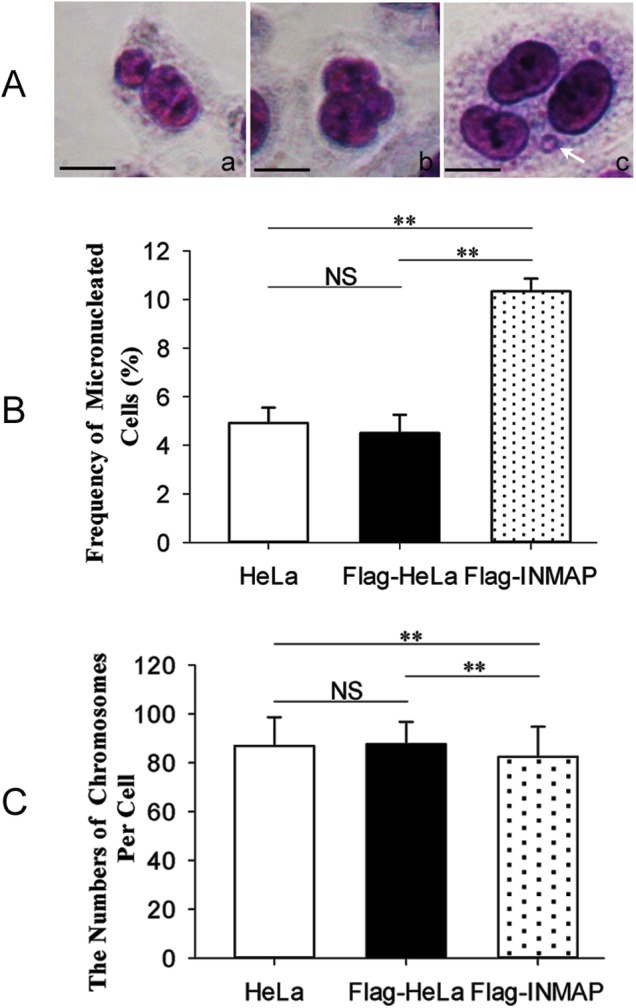
*INMAP* overexpression induces genomic instability in HeLa cells. (A, B). The effect of *INMAP* overexpression on the induction of micronuclei. (A). Mono-, bi- and tri-nucleated cells, stained with Giemsa. Arrowhead indicates micronucleus. (B). The frequency of micronucleated cells in Flag-INMAP cells (n = 400) is greater than in Flag-HeLa or HeLa. (C). The numbers of chromosomes were determined in *INMAP*-overexpressing cells (n = 100). Scale bars, 5 μm.

Micronuclei (MNi), small nucleus-like interphase structures in the cytoplasm of cells (Fig. 3A), have been used extensively as a biomarker to identify genomic instability and DNA damage events. To detect whether overexpression of *INMAP* has an effect on micronuclei formation, we analysed 400 cells per condition. As shown in Fig. 3B, the frequency of micronucleated cells is 4.92% ± 0.63% (HeLa), 4.50% ± 0.75% (Flag-HeLa) and 10.33% ± 0.52% (Flag-INMAP), respectively. This value is significantly higher in Flag-INMAP cells compared with HeLa cells (*P* = 0.00) and Flag-HeLa cells (*P* = 0.00). Clearly, increasing the expression of *INMAP* promoted the formation of micronuclei in HeLa cells.

Micronuclei may contain either chromosomes or chromosomal fragments [17, 18]. Since abnormal expression of *INMAP* increases micronuclei in HeLa cells, we assayed the change in chromosomes. As shown in Fig. 3C, the number of chromosomes from Flag-INMAP cells is 82.53 ± 12.30, while those from HeLa and Flag-HeLa cells are 86.95 ± 11.72 and 87.67 ± 9.18 (both *P* < 0.01, compared to Flag-INMAP cells), respectively. These results indicated that chromosome numbers decrease in cells abnormally expressing *INMAP*. Therefore, INMAP is an important protein for maintaining stable numbers of chromosomes in mitosis, ensuring genomic integrity and stability.

### Overexpression of *INMAP* induced DNA damage in HeLa cells

**Figure 4:**
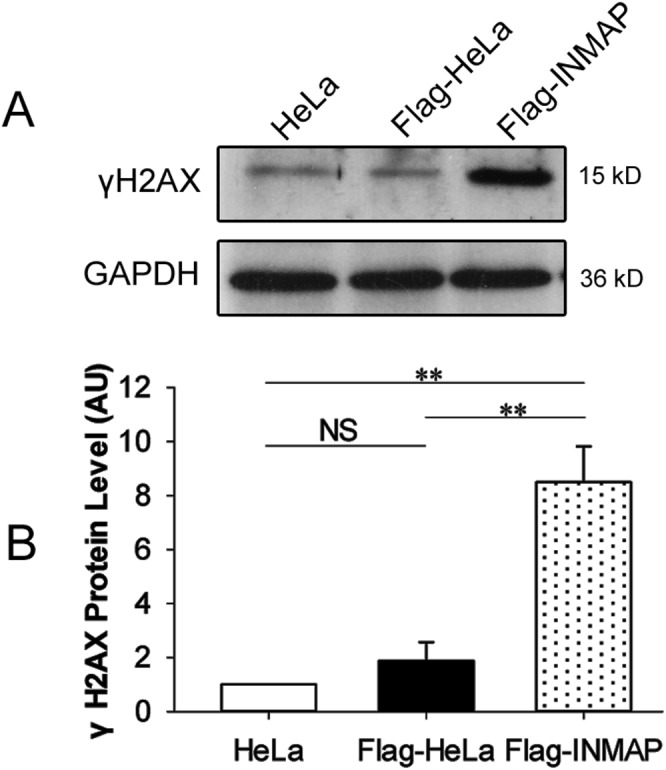
*INMAP* overexpression induces DNA damage in HeLa cells. (A). Western blot analysis of HeLa, Flag-HeLa and Flag-INMAP cells with anti-γH2AX and anti-GAPDH antibodies. (B). Each band quantification was analysed with Image J software. Data are representative of three independent experiments. AU, arbitrary unit.

To clarify whether overexpressing *INMAP* induces DNA damage in HeLa cells, we analysed the level of γH2AX, a sensitive marker for detecting DNA double-strand breaks (DSBs) in the DNA-damage response [19], using Western blotting. As shown in Fig. 4, we found *γH2AX* expression markedly upregulated in cells overexpressing INMAP. This result confirmed that overexpressing INMAP induces DSBs in HeLa cells. Clearly, INMAP has a significant function in maintaining genomic DNA completeness.

### Overexpression of *INMAP* suppressed LDH activity

LDH is an important enzyme converting pyruvate to lactate under hypoxic conditions and, as a vital biochemical marker to assess the activity of cancer cells in energy metabolism, is believed to play an important role in the development and progression of malignancies [20]. To determine whether overexpressing INMAP affects LDH activity in HeLa cells, we analysed the levels of LDH isoenzymes. We detected five electrophoretic bands (LDH1~LDH5) distributed in HeLa cells (S2A Fig.); the activity of LDH1 is higher in *INMAP*-overexpressing cells than in HeLa cells (*P* = 0.00) or Flag-HeLa cells (*P* = 0.00), whereas the activity of LDH5 is lower in *INMAP-*overexpressing cells than in HeLa cells (*P* = 0.01) or Flag-HeLa cells (*P* = 0.05) (S2B Fig.). Studies have shown that LDH5 is mainly overexpressed in cancer cells and is linked with tumour necrosis and increases in tumour size, whereas LDH1 is more widely expressed and often downregulated in cancer cells compared with normal tissues [21, 22]. Our study suggested that overexpression of *INMAP* suppresses LDH activity via a biochemical criterion.

### INMAP interacts with p21

**Figure 5:**
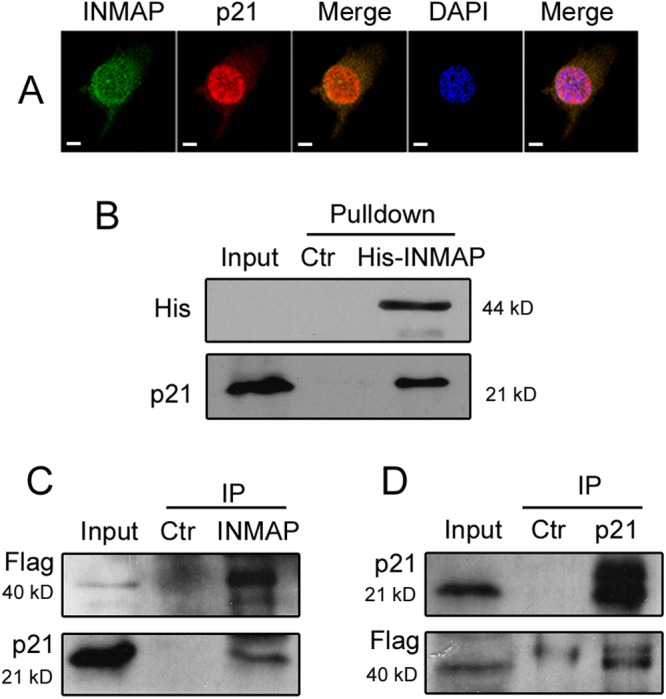
INMAP interacts with p21. (A). Colocalisation of INMAP and p21 in *INMAP* overexpression HeLa cells (Flag-INMAP). Cells were double-stained with mouse monoclonal anti-Flag antibody (green) and rabbit monoclonal anti-p21 antibody (red), and stained cells were analysed with IIF. Where green and red signals overlap (Merge), a yellow pattern is observed, indicating the colocalisation of INMAP and p21. Scale bars, 5 μm. (B). Pulldown assay of INMAP and p21. Expression vector pET30a-*INMAP* was transformed into *Escherichia coli* BL21. The purified His-INMAP fusion protein was added into HeLa cell extracts, incubated, and recovered on beads. pET30a (+) was a control. The Pulldown product was analysed by Western blotting. (C, D). CoIP assay of INMAP with p21. The proteins of HeLa cells that expressed Flag-INMAP were extracted and immunoprecipitated with the monoclonal Flag antibody (C) or p21 antibody (D). The same amount of purified mouse IgG (C) or rabbit IgG (D) was used in control samples. The immunoprecipitation (IP) result was analysed with SDS-PAGE and Western blot with anti-p21 or anti-Flag antibody.

p53 is involved in various cellular processes that regulate the cell cycle and apoptosis under conditions of DNA damage or genomic aberration. Considering the association with genomic integrity, we examined the interaction of INMAP and associated proteins (p53, p21, and PCNA) by CoIP analysis, intending to determine whether INMAP acts through the p53-mediated signalling pathway. As shown in Fig. 5B-D, we confirmed the interaction of INMAP and p21 by Pulldown and CoIP, thereby revealing that INMAP and p21 can form a complex, supporting their direct interaction. We further demonstrated that INMAP and p21 colocalize as distinct dots in the nucleus and cytoplasm by IIF and confocal microscopy.

### Effects of *INMAP* overexpression on the expression of genes related to proliferation and apoptosis

**Figure 6:**
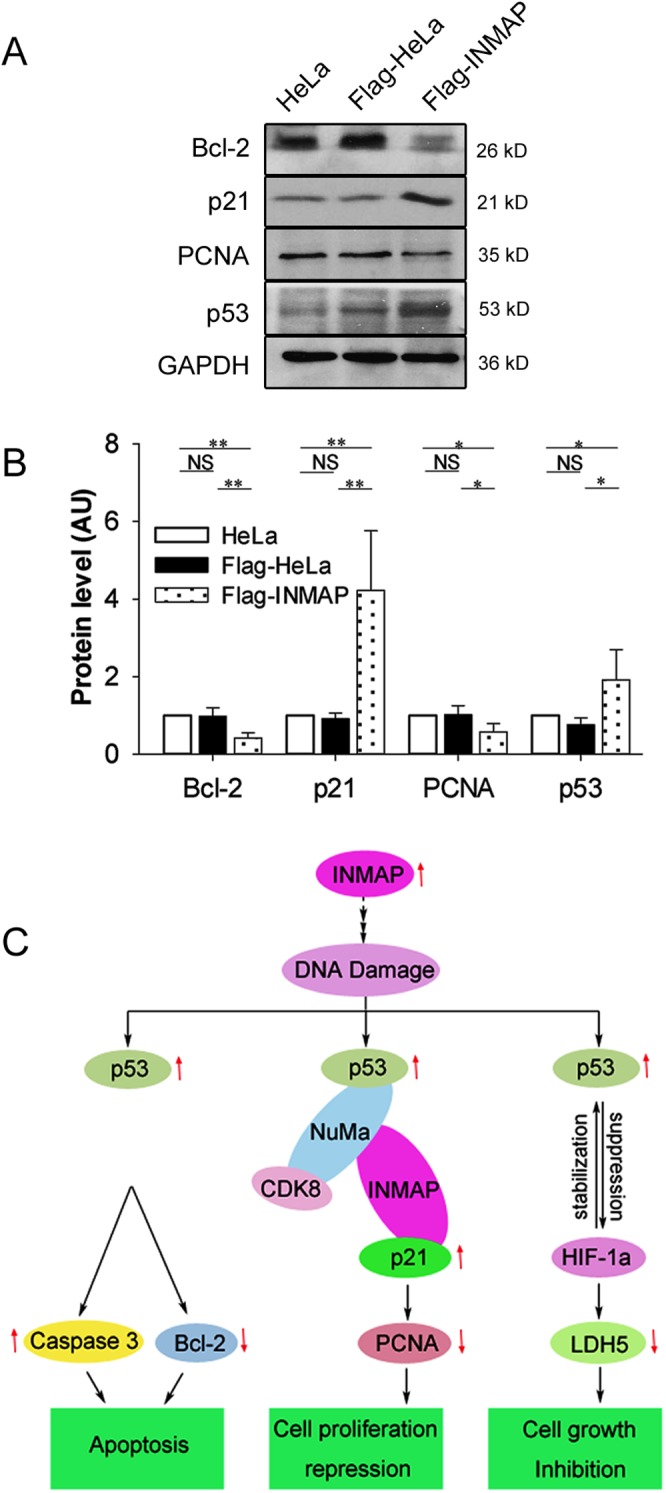
Effects of *INMAP* overexpression on the expression of genes related to proliferation and apoptosis. (A). Western blot analysis of the three groups of cells (HeLa, Flag-HeLa, and Flag-INMAP) with anti-Bcl-2, anti-p53, anti-p21 and anti-PCNA antibodies. Data shown are representative of three independent experiments. (B). Band quantification was analysed with Image J software. (C). A schematic model illustrating the proposed mechanisms of *INMAP*-overexpression-induced DNA damage and apoptosis through p53-dependent pathways in transformed cells.

To explain the molecular mechanism by which *INMAP* overexpression affects cell proliferation and apoptosis, we examined several key molecules concerned with tumour progression in *INMAP*-overexpressing and control cells. As shown in Fig. 6A, we found that p53 and p21 were markedly upregulated, whereas PCNA was downregulated, suggesting that overexpression of *INMAP* leads to cell-cycle arrest. To further verify the role of p21 in cell death under *INMAP* overexpression, we detected an apoptosis factor, Bcl-2. The result showed that the expression of *Bcl-2* decreases when *INMAP* expressed excessively, consistent with our previous finding that caspase 3 increases under this circumstance [10]. These results suggest that the high level of INMAP exerts the inhibitory function on HeLa cells by regulating cell proliferation and apoptosis and causes accumulation of the p53 protein and activation of the p53-dependent pathways (Fig. 6B).

## Discussion

In this study, we tested the tumour-suppressing function of overexpressed *INMAP* through both *in vitro* and tumour bearing nude mouse *in vivo* assays. Our results show that the *INMAP* expression transgene in HeLa cells dramatically inhibits both anchorage-independent cell growth in soft agar cultures and anchorage-dependent cell growth in monolayer cultures (Fig. 1A and C), whereas it has little effect on implanted cell proliferation in nude mice. Compared to control groups, we found the average values of tumour size and tumour weight were reduced in nude mice injected with *INMAP* overexpressed cells, but there was no significant difference (*P* > 0.05) (Fig. 2B and C). However, intriguingly, we found that the cell density decreased and necrosis increased in *INMAP*-overexpressing HeLa xenografts by H&E staining (Fig. 2F), PCNA and Bcl-2 are downregulated by Western blot assay. We deduce that the necrosis in xenografts may be a post-apoptotic result induced by the high level of INMAP. These findings suggested that *INMAP* overexpression perturbs cell division, and the *INMAP* gene may have an inhibitory effect on cell proliferation in HeLa cells when its expression aberrantly increased, through cell-cycle arrest and apoptosis signalling pathways. To elucidate whether INMAP is commonly a suppressor in cancer development when its expression is altered requires further investigation of the levels of INMAP in diverse tumour tissues in future studies.

Our previous study revealed that *INMAP* overexpression in HeLa cells causes a decrease in DNA content [1]. Consistently, we detected that the frequencies of MNi and the level of γH2AX were significantly increased in *INMAP* overexpressed HeLa cells in the present study (Fig. 3B; Fig. 4). These results suggested that the expression of the *INMAP* transgene in HeLa cells has a substantial impact on genomic stability. MNi mainly originate from incorrectly aligned chromosomes in metaphase, lagging chromosomes, nuclear buds in S phase and broken chromosome bridges in later mitotic stages [23–25]. Clearly, the increase of MNi (Fig. 3C) is consistent with the decrease of chromosome number in *INMAP*-overexpressing HeLa cells as well as the upregulation γH2AX. A study on the fate of micronucleated cells has revealed a positive association between micronuclei and cell apoptosis, and the frequency of apoptotic cell death is much higher among cells bearing micronuclei compared with cells bearing normal nuclei [24]. Based on these results, we deduce that the overexpression of *INMAP* in HeLa cells may induce DNA damage, cause the formation of MNi, and consequently result in growth inhibition and apoptosis.

LDH serves as an important biochemical tumour marker to assess the activity and the grade of malignancy, since hypoxia is a basic feature in tumours. Five isoforms of LDH (LDH 1~5) have been identified [20, 26, 27]. The LDH5 isoenzyme, encoded by the *LDHA* gene, is the most important for mediating the conversion of pyruvate to lactic acid in glycolysis [28]. Several reports have shown that LDH5 is related to tumour formation, lymph node and distant metastases; its high level is linked with tumour necrosis and increased tumour size [22, 26, 29, 30]. In contrast, LDH1 is downregulated in tumours and mediates the conversion of pyruvate to acetyl-CoA, which enters the citric acid cycle [29, 31]. We found that the overexpression of *INMAP* decreases the LDH5 activity and increases the LDH1 activity in HeLa cells, suggesting that an excessive level of INMAP reduces energy for cell growth in glycolysis, and ultimately suppresses cellular malignancy. Inhibition of glycolysis in cancer cells is a novel strategy to cure cancers [32], and the corresponding exploration of INMAP functional course may indicate its utility as a target.

Obviously, associating INMAP-induced DNA damage, growth inhibition and apoptosis with the mechanism of INMAP in suppressing tumour growth is worthwhile. Cell-cycle arrest and apoptosis are two major outcomes of p53 activation, which contributes to validating the DNA damage response signalling pathway [33, 34]. Cell-cycle arrest allows DNA repair to take place before replication occurs, thereby maintaining genomic integrity. Thus, apoptosis results in the elimination of irreparably damaged cells [35]. Recent study showed that NuMA is a key factor in responding to DNA damage through the p53 pathway. NuMA might play the role of a scaffold protein to recruit the mediator complex, binding to p53 and CDK8 to promote the transcription of pro-arrest genes and cell-cycle arrest [11]. As a flexible component of the mediator complex, CDK8 is responsible for bridging between specific and general transcription factors. Suppression of *CDK8* expression inhibits the proliferation of colon cancer cells, which were originally characterised by their high level of CDK8 expression [36]. CDK8 is recruited to the p21 promoter to activate transcription in response to DNA damage [37]. Our previous study revealed that overexpression of *INMAP* inhibits p53 and AP-1 activity in HEK293T cells [10], showing that its function may be associated with the p53 pathway. We also identified that INMAP interacts with NuMA, a selective inducing factor of p53 target gene, in HeLa cells [1].

More interestingly, we detected by Pulldown, CoIP and IIF assays that INMAP interacts with p21 in HeLa cells (Fig. 5). Subsequently, the gene expression levels related to cell-cycle arrest and apoptosis were analysed by Western blot, revealing that *INMAP* overexpression in HeLa cells increases p53, p21 and caspase 3 [10] protein levels, whereas it decreases PCNA and Bcl-2 protein levels (Fig. 6A). Clearly, INMAP has the ability to activate gene transcription related to cell-cycle arrest and apoptosis, and the mechanism of INMAP function related to p53 pathway may be as follows: Overexpression of *INMAP* in HeLa cells results in DNA damage and p53 activation. The active p53 regulates various genes to inhibit cell proliferation, which causes cell-cycle arrest, apoptosis and the change of the intracellular oxygen level. INMAP combines with NuMA to form a complex, and it might assist NuMA to recruit CDK8 to the promoter region of the *p21* gene in cell-cycle arrest. In addition, INMAP interacts with p21, which may directly control the expression of *p21*, affect the downstream gene *PCNA* and inhibit DNA synthesis. Bcl-2 and caspase 3 are key factors in the p53-mediated apoptotic pathway [34, 38]. Bcl-2 downregulation and caspase 3 upregulation coincide in the apoptotic pathway mediated by activated p53, a “classical” signalling pathway.

Studies have shown that p53 and its interactional factor, HIF-1α (hypoxia inducible transcription factor 1α), are major regulators of the cellular response to hypoxia [39–42]. Interestingly, the high ratio of p53 transcription is a marker of advanced malignancy. The function of HIF-1α is to maintain p53 stabilisation. When cancer cells have lost p53 function, they shift a balance from p53 to HIF transcriptional regulation. However, p53 inhibits *HIF-1α* expression [43, 44]. Additionally, HIF-1α directly controls the expression of *LDHA*; HIF-1α and LDH5 are commonly expressed at high levels in cancer cells [45, 46]. We detected that *INMAP* overexpression decreases LDH5 activity, and it may be deduced that INMAP may regulate the p53-mediated HIF-1α pathway. *p53* overexpression may inhibit *HIF-1α* expression, and HIF-1α might then suppress *LDHA* expression.

In this study, we found that overexpression of *INMAP* in HeLa cells causes DNA damage/genomic instability and apoptosis, thereby suppressing tumour growth both *in vitro* and *in vivo*. Moreover, a high INMAP level activates key genes associated with DNA damage and apoptosis through p53-medicated signalling pathways. The exact mechanism of p53 signalling pathways triggered by excessive expression of *INMAP* remains to be further studied. These results underscore the crucial role of INMAP in carcinogenesis and tumour growth.

## Supporting Information

S1 Fig Overexpression of INMAP in HeLa cell. (A). Single cloned cells stably expressed Flag-INMAP protein were identified with anti-INMAP polyclonal antibody and anti-Flag antibody.
(B). Band quantification was analysed with Image J software and SPSS 19.0 software. Data are representative of three independent experiments. AU, arbitrary unit.
(TIF)

S2 Fig Analysis of LDH isoenzyme spectrum and activity. (A). LDH isoenzyme pattern was analysed with 7.5% PAGE in HeLa cells. Bands show LDH1, LDH2, LDH3, LDH4 and LDH5, respectively (from anode to cathode).
(B). Analysis of LDH isoenzyme activity. Each band quantification of LDH isoenzyme was analysed with Image J software and SPSS 19.0 software.
(TIF)
