# Supplementary figures and images for: INMAP Overexpression Inhibits Cell Proliferation, Induces Genomic Instability and Functions through p53/p21 Pathways
Source: PLoS One. 2015 Jan 30;10(1):e0115704. doi: 10.1371/journal.pone.0115704 (PMC4312054; doi:10.1371/journal.pone.0115704)

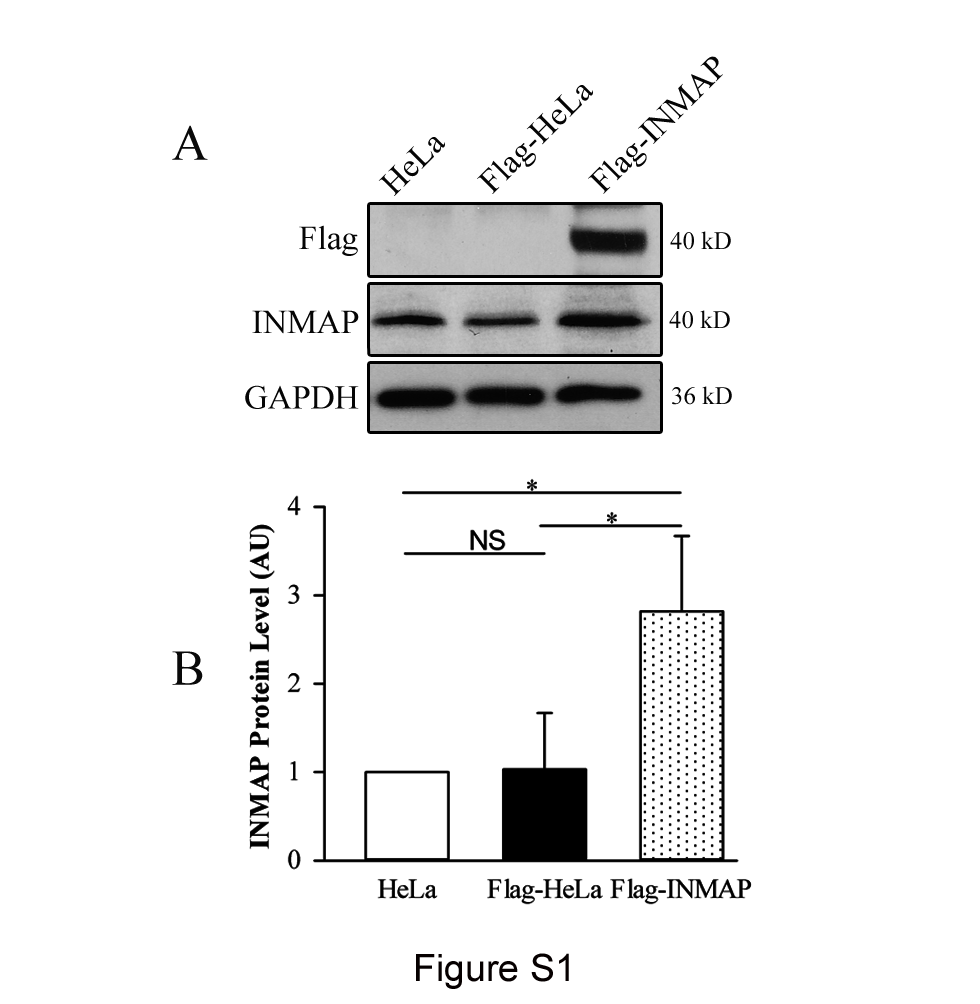

Supplement: S1 Fig — (A). Single cloned cells stably expressed Flag-INMAP protein were identified with anti-INMAP polyclonal antibody and anti-Flag antibody. (B). Band quantification was analysed with Image J software and SPSS 19.0 software. Data are representative of three independent experiments. AU, arbitrary unit. (TIF) [file pone.0115704.s001.tif]

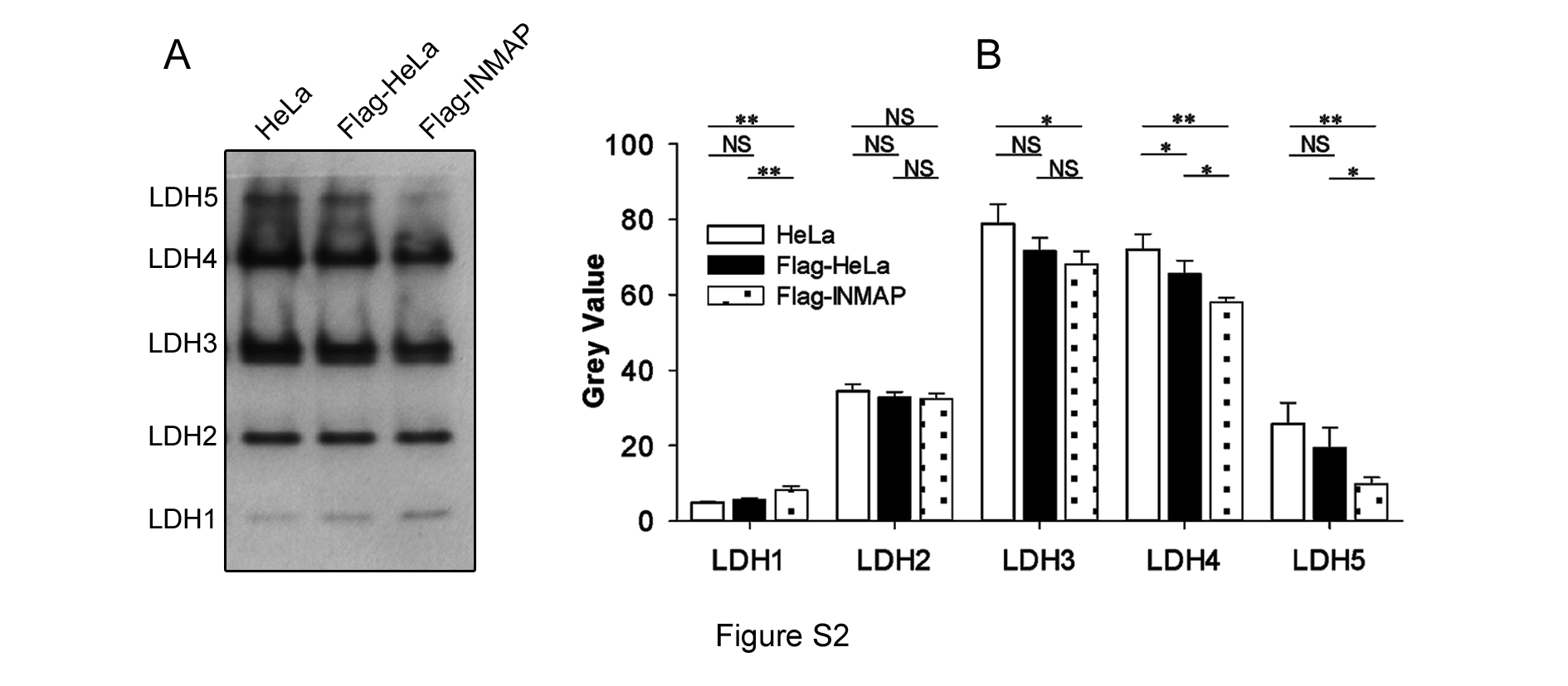

Supplement: S2 Fig — (A). LDH isoenzyme pattern was analysed with 7.5% PAGE in HeLa cells. Bands show LDH1, LDH2, LDH3, LDH4 and LDH5, respectively (from anode to cathode). (B). Analysis of LDH isoenzyme activity. Each band quantification of LDH isoenzyme was analysed with Image J software and SPSS 19.0 software. (TIF) [file pone.0115704.s002.tif]
